# Mortality and lung function decline in patients who develop chronic pulmonary aspergillosis after lung cancer surgery

**DOI:** 10.1186/s12890-022-02253-y

**Published:** 2022-11-22

**Authors:** Bo-Guen Kim, Yong Soo Choi, Sun Hye Shin, Kyungjong Lee, Sang-Won Um, Hojoong Kim, Yeong Jeong Jeon, Junghee Lee, Jong Ho Cho, Hong Kwan Kim, Jhingook Kim, Young Mog Shim, Byeong-Ho Jeong

**Affiliations:** 1 Division of Pulmonary and Critical Care Medicine, Department of Medicine, Samsung Medical Center, Sungkyunkwan University School of Medicine, Irwon-ro 81, Gangnam-gu, Seoul, 06351 Republic of Korea; 2 Department of Thoracic and Cardiovascular Surgery, Samsung Medical Center, Sungkyunkwan University School of Medicine, Seoul, Republic of Korea

**Keywords:** Lung cancer, Surgery, Chronic pulmonary aspergillosis, Respiratory function tests, Mortality

## Abstract

**Background:**

Lung cancer surgery is reported as a risk factor for chronic pulmonary aspergillosis (CPA). However, limited data are available on its clinical impact. We aimed to determine the effect of developed CPA after lung cancer surgery on mortality and lung function decline.

**Methods:**

We retrospectively identified the development of CPA after lung cancer surgery between 2010 and 2016. The effect of CPA on mortality was evaluated using multivariable Cox proportional hazard analyses. The effect of CPA on lung function decline was evaluated using multiple linear regression analyses.

**Results:**

During a median follow-up duration of 5.01 (IQR, 3.41–6.70) years in 6777 patients, 93 developed CPA at a median of 3.01 (IQR, 1.60–4.64) years. The development of CPA did not affect mortality in multivariable analysis. However, the decline in forced vital capacity (FVC) and forced expiratory volume in 1 second (FEV_1_) were greater in patients with CPA than in those without (FVC, − 71.0 [− 272.9 to − 19.4] vs. − 10.9 [− 82.6 to 57.9] mL/year, *p* < 0.001; FEV_1_, − 52.9 [− 192.2 to 3.9] vs. − 20.0 [− 72.6 to 28.6] mL/year, *p* = 0.010). After adjusting for confounding factors, patients with CPA had greater FVC decline (β coefficient, − 103.6; 95% CI − 179.2 to − 27.9; *p* = 0.007) than those without CPA. However, the FEV_1_ decline (β coefficient, − 14.4; 95% CI − 72.1 to 43.4; *p* = 0.626) was not significantly different.

**Conclusion:**

Although the development of CPA after lung cancer surgery did not increase mortality, the impact on restrictive lung function deterioration was profound.

**Supplementary Information:**

The online version contains supplementary material available at 10.1186/s12890-022-02253-y.

## Background

Lung cancer is the most common cause of cancer death globally, and the incidence is increasing [1, 2]. In recent decades, advances in surveillance and treatment strategies have improved the long-term survival rates of lung cancer patients [3, 4]. This clinical success has been observed mainly in early-stage lung cancer, which can be surgically resected, rather than advanced lung cancer [5, 6]. As long-term follow-up of lung cancer patients after surgery has become more attainable, the frequency of chronic pulmonary infections has increased [7].

Chronic pulmonary aspergillosis (CPA) is a slowly destructive pulmonary disease in patients with pre-existing chronic pulmonary disease [8–11]. Colonization of *Aspergillus* species in the residual cavities of the lung parenchyma is a sequela of chronic pulmonary disease and is responsible for CPA [12]. CPA was relatively overlooked for a long time by physicians but has recently been recognized as a serious global health burden [8, 11]. Previous studies have reported that lung cancer surgery is one of the risk factors for CPA [7, 13]. These studies showed that development of CPA was closely related to underlying pulmonary disease, postoperative pulmonary complication (PPC), and neoadjuvant/adjuvant therapy in lung cancer patients who underwent lung cancer surgery [7, 13]. In addition, the incidence of CPA increased as the postoperative survival time of patients lengthened.

However, limited data are available regarding the clinical impacts of the development of CPA after lung cancer surgery. Therefore, we aimed to investigate the clinical characteristics of CPA that occur after lung cancer surgery especially focusing on mortality and lung function decline.

## Methods

### Study population and data collection

Using the Lung Cancer Surgery Registry at Samsung Medical Center (a 1979-bed referral hospital in South Korea), we retrospectively identified the development of CPA after lung cancer surgery in patients with non-small cell lung cancer (NSCLC) between January 2010 and December 2016. This study included previously published data from patients who underwent lung cancer surgery between January 2010 to December 2013 [7]. Patients with CPA at the time of surgery were excluded.

We used the electronic medical records to gather the following information: patient-related factors including age, sex, body mass index (BMI), smoking history, underlying pulmonary diseases, other comorbidities, and pulmonary function test results; cancer-related factors including histologic type, location of the tumor, and clinical/pathological stage; cancer treatment-related factors including neoadjuvant or adjuvant treatments used, surgical approach, extent of surgical resection, and PPC within 30 days after surgery [7]; CPA-related factors such as confirmation method of *Aspergillus* species and antifungal drug used. The tumor was staged using the 7th edition of the American Joint Committee on Cancer [14]. PPC was defined as development of intrathoracic complications during hospital stay or readmission within 30 days after surgery [15]. Patient follow-up data were last updated in August 2021.

This study obtained approval from Samsung Medical Center Institutional Review Board (SMC IRB no. 2021-10-046) to review and publish information from patient records. The requirement for informed consent was waived by SMC IRB due to the retrospective nature of the study.

### Diagnosis of CPA

After surgical resection, most patients were routinely followed for at least 5 years by thoracic surgeons. Pulmonologists jointly followed up patients with pre-existing or newly developed pulmonary diseases [7]. When CPA was suspected, patients were referred to pulmonologists, and further diagnostic tests were performed. CPA was diagnosed according to the European Society for Clinical Microbiology and Infectious Diseases/European Respiratory Society (ERS) criteria: (1) compatible clinical symptoms; (2) serological or microbiological evidence: positive serum *Aspergillus* precipitin test (*Aspergillus fumigatus* IgG ELISA kit; IBL International, Hamburg, Germany); isolation of *Aspergillus* species from a respiratory specimen, or histologic confirmation; (3) compatible radiological findings with overt progression; and (4) exclusion of alternative diagnosis [11, 16].

### Spirometry and annual lung function decline

Spirometry was performed using a Vmax 22 system (SensorMedics, Yorba Linda, CA, USA) following the American Thoracic Society/ERS guidelines [17]. Absolute values of forced vital capacity (FVC) and forced expiratory volume in one second (FEV_1_) were obtained from a pre-bronchodilator test, and the predicted percentage values (% predicted) for FVC and FEV_1_ were calculated using the equation obtained by analyzing the representative value of the Korean population [18].

An obstructive pattern was defined as FEV_1_/FVC < 70% and predicted FEV_1_ < 80%, and a restrictive pattern was defined as FEV_1_/FVC ≥ 70% and predicted FVC < 80%. Patients with FEV_1_/FVC ≥ 70% and predicted FVC ≥ 80% were classified as having a normal pattern. Patients with FEV_1_/FVC < 70% and predicted FEV_1_ ≥ 80% were also classified as having a normal pattern [19]. Annual lung function decline rates (ml/year) were calculated in patients who had a pulmonary function test (PFT) at least 3 months after surgery when lung function was considered to have recovered [20]. To calculate the annual rate of decline, only patients who had at least two PFT results that were at least 6 months apart were counted. For patients with CPA, only those with follow-up PFT results after the diagnosis of CPA were included in the analysis. The lung function decline was calculated as [(last FVC [or FEV_1_]) – (FVC [or FEV_1_] at baseline)]/follow-up duration (years) [19, 21]. Rapid progression of FVC (or FEV_1_) decline was defined as ≥ 40 mL loss per year in FVC (or FEV_1_) [22, 23].

### Statistical analyses

All data are expressed as the median (interquartile range, IQR) for continuous variables, and as the numbers (%) for categorical variables. Categorical variables were compared using Pearson χ^2^ test or Fisher’s exact test, and continuous variables were compared using Mann–Whitney U test. The Kaplan–Meier method was used to estimate the cumulative incidence of CPA and overall survival (OS) after the lung cancer surgery. The log-rank test was used to compare survival according to the development of CPA [7].

The effect of CPA on mortality was evaluated using multivariable Cox proportional hazard analyses. Three models were used: model 1 was adjusted for clinical baseline characteristics; model 2 was adjusted for variables with lung cancer-related factors; model 3 was adjusted for all the preceding variables.

The effect of CPA on lung function decline was evaluated using multiple linear regression analyses. Five models were constructed: model 1 was adjusted for baseline FVC [L] in FVC decline and for baseline FEV_1_ [L] in FEV_1_ decline; model 2 was adjusted for selected variables with *p* < 0.20 in univariate analyses with consideration of multicollinearity; model 3 was adjusted for variables that were generally considered to influence the decline of lung function; model 4 was adjusted for variables related to lung cancer treatment and variables considered to be related to the development of CPA; and, finally, model 5 was adjusted for all the preceding variables.

All tests were two-sided, and a *p* value < 0.05 was considered significant. We used PASW Statistics 27 (SPSS Inc., Chicago, IL, USA) for analysis.

## Results

### Study population and development of CPA after lung cancer surgery

**Fig. 1:**
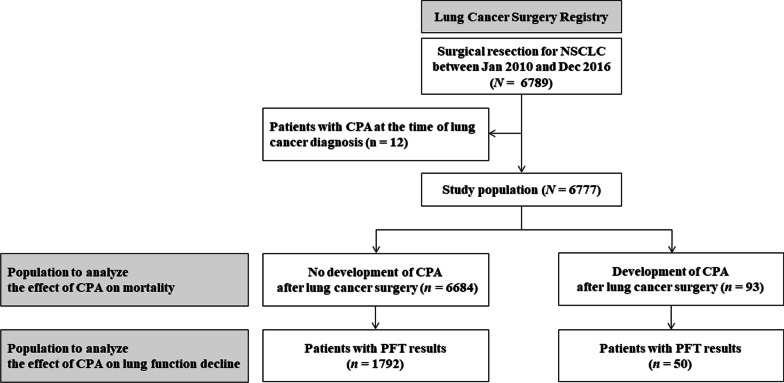
Flow diagram of the study population. *NSCLC* Non-small cell lung cancer, *CPA* Chronic pulmonary aspergillosis, *PFT* Pulmonary function test

**Fig. 2:**
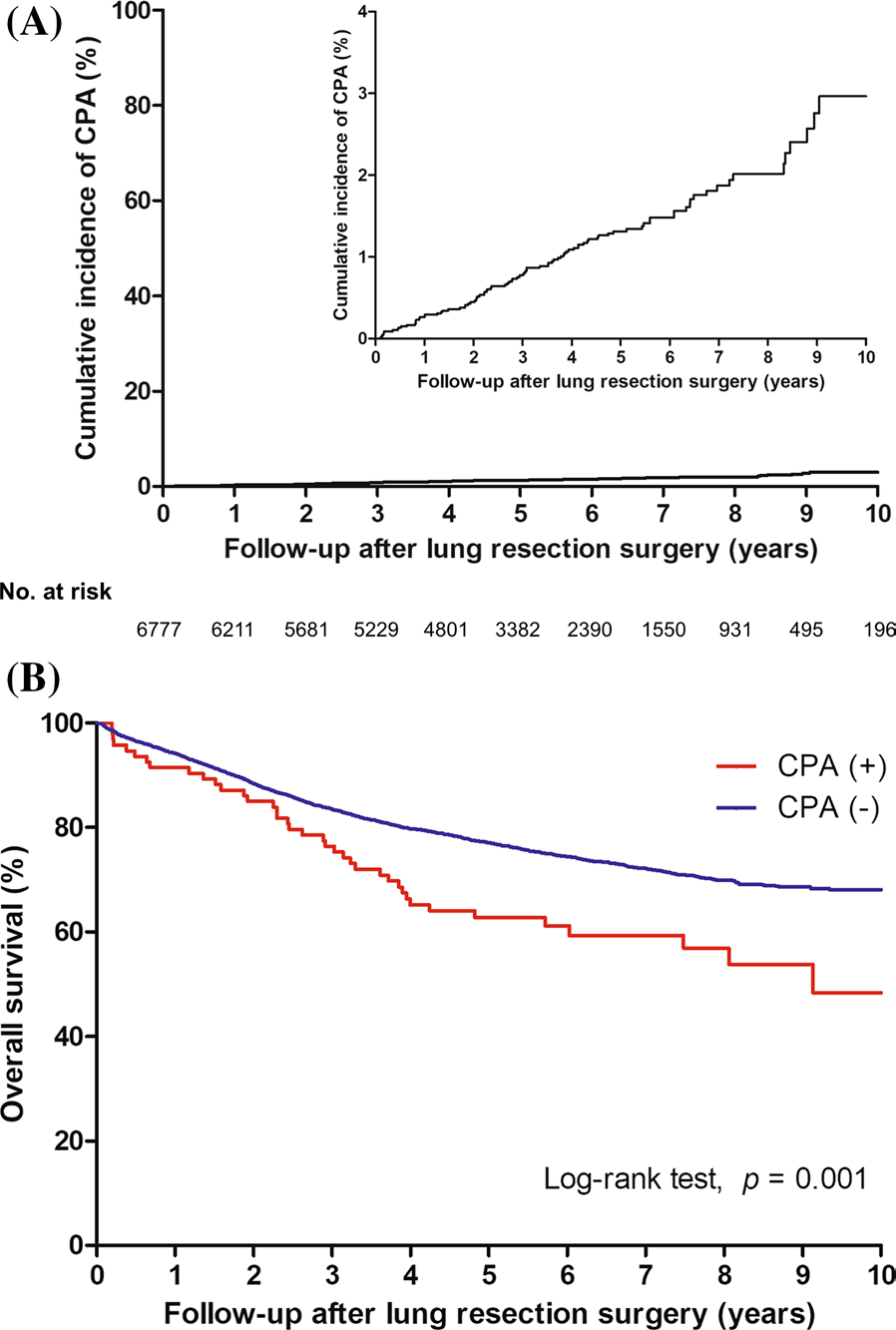
**A** Cumulative incidence of chronic pulmonary aspergillosis after lung cancer surgery. **B** Overall survival according to the development of CPA. *CPA* Chronic pulmonary aspergillosis

In 6789 patients who underwent lung cancer surgery for NSCLC, 12 who were diagnosed with CPA and lung cancer at the same time or had a previous history of CPA were excluded from this study (Fig. 1). Finally, of 6777 patients, 93 developed CPA at a median of 3.01 (IQR, 1.60–4.64) years after lung cancer surgery. The cumulative incidences of CPA after lung cancer surgery were 0.3%, 0.8%, 1.3%, and 3.0% at 1, 3, 5, and 10 years, respectively (Fig. 2 A).

The median age of the study population was 63 years (IQR, 56–69 years), and 61.3% were male (Table 1). More than half of the patients (57.8%) were either ex- or current smokers. Most of the patients (69.0%) were in clinical stage I, and adenocarcinoma (70.5%) was the most common tumor histology. Neoadjuvant or adjuvant therapy was received by 647 (9.5%) and 1845 (27.4%) patients, respectively. Video-assisted thoracoscopic surgery was performed in 62.4%, and most patients (76.1%) underwent lobectomy. PPCs within 30 days occurred in 17.1% of patients.

**Table 1:** Baseline characteristics and treatment profile of patients with NSCLC according to the development of CPA after lung resection

Variables	Total (n = 6777)	CPA (–) (n = 6684)	CPA (+) (n = 93)	*P*
*Baseline characteristics*				
Age, years	63 (56–69)	63 (56–70)	63 (57–69)	0.731
Sex, male	4151 (61.3)	4068 (60.9)	83 (89.2)	< 0.001
BMI, kg/m^2^	23.9 (22.0–25.8)	23.9 (22.0–25.8)	22.5 (20.5–24.4)	< 0.001
Smoking status (n = 6775)				< 0.001
Never smoker	2857 (42.2)	2844 (42.6)	13 (14.0)	
Ex-smoker	2117 (31.2)	2077 (31.1)	40 (43.0)	
Current smoker	1801 (26.6)	1761 (26.4)	40 (43.0)	
Pack-years (n = 3868)	30 (20–45)	30 (20–45)	40 (30–60)	0.003
*Comorbidity*				
Underlying pulmonary disease				
COPD/Asthma	1864 (27.5)	1822 (27.3)	42 (45.2)	< 0.001
Previous history of PTB	754 (11.1)	739 (11.1)	15 (16.1)	0.133
Interstitial lung disease	77 (1.1)	73 (1.1)	4 (4.3)	0.021
DM	1074 (15.8)	1059 (15.8)	15 (16.1)	0.940
Previous history of malignancy	939 (13.9)	926 (13.9)	13 (14.0)	0.972
Chronic heart disease	474 (7.0)	470 (7.0)	4 (4.3)	0.305
Cerebrovascular disease	388 (5.7)	384 (5.7)	4 (4.3)	0.552
Chronic renal disease	95 (1.4)	95 (1.4)	0 (0.0)	0.643
Clinical stage at diagnosis				< 0.001
Stage I	4678 (69.0)	4644 (69.5)	34 (36.6)	
Stage II	1176 (17.4)	1159 (17.3)	17 (18.3)	
Stage III	858 (12.7)	817 (12.2)	41 (44.1)	
Stage IV	65 (1.0)	64 (1.0)	1 (1.1)	
Tumor histology				< 0.001
Adenocarcinoma	4780 (70.5)	4734 (70.8)	46 (49.5)	
Squamous cell carcinoma	1588 (23.4)	1552 (23.2)	36 (38.7)	
Others^a^	409 (6.0)	398 (6.0)	11 (11.8)	
Lobar location of cancer				0.516
Right	3940 (58.1)	3889 (58.2)	51 (54.8)	
Left	2837 (41.9)	2795 (41.8)	42 (45.2)	
*Treatment profile for NSCLC*				
Neoadjuvant treatment				< 0.001
No	6130 (90.5)	6070 (90.8)	60 (64.5)	
Yes	647 (9.5)	614 (9.2)	33 (35.5)	
CCRT	561 (8.3)	530 (7.9)	31 (33.3)	< 0.001
Chemotherapy	79 (1.2)	77 (1.2)	2 (2.2)	0.296
Radiotherapy	7 (0.1)	7 (0.1)	0 (0.0)	> 0.999
Surgical approach				< 0.001
VATS	4229 (62.4)	4210 (63.0)	19 (20.4)	
Thoracotomy	2548 (37.6)	2474 (37.0)	74 (79.6)	
Types of surgical resection				< 0.001
Sublobar resection	1126 (16.6)	1120 (16.8)	6 (6.5)	
Lobectomy	5158 (76.1)	5083 (76.0)	75 (80.6)	
Bilobectomy	262 (3.9)	251 (3.8)	11 (11.8)	
Pneumonectomy	231 (3.4)	230 (3.4)	1 (1.1)	
Pathologic stage (n = 6712^b^)				0.004
Stage I	4327 (64.5)	4286 (64.7)	41 (47.1)	
Stage II	1256 (18.7)	1235 (18.6)	21 (24.1)	
Stage III	1052 (15.7)	1028 (15.5)	24 (27.6)	
Stage IV	77 (1.1)	76 (1.1)	1 (1.1)	
Postoperative pulmonary complication within 30 days ^c^	1161 (17.1)	1129 (16.9)	32 (34.4)	< 0.001
Adjuvant treatment (n = 6727 ^d^)				< 0.001
No	4882 (72.6)	4834 (72.9)	48 (51.6)	
Yes	1845 (27.4)	1800 (27.1)	45 (48.4)	
CCRT	339 (5.0)	328 (4.9)	11 (11.8)	0.007
Chemotherapy	1184 (17.6)	1164 (17.5)	20 (21.5)	0.319
Radiotherapy	322 (4.8)	308 (4.6)	14 (15.1)	< 0.001

Data are presented as n (%) or the median (interquartile range)

*NSCLC* Non-small cell lung cancer, *CPA* Chronic pulmonary aspergillosis, *BMI* Body mass index, *COPD* Chronic obstructive pulmonary disease, *PTB* Pulmonary tuberculosis, *DM* Diabetes mellitus, *CCRT* Concurrent chemoradiotherapy, *VATS* Video-assisted thoracoscopic surgery, *ypCR* Pathological complete response after neoadjuvant treatment, *ARDS* Acute respiratory distress syndrome

^a^Includes large cell neuroendocrine carcinoma, adenosquamous carcinoma, pleomorphic carcinoma, adenoid cystic carcinoma, mucoepidermoid carcinoma, epithelial myoepithelial carcinoma, and carcinoid tumors

^b^Except for 65 patients with ypCR.

^c^Pneumothorax/prolonged air leak (n = 577), ARDS/respiratory failure required mechanical ventilation (n = 322), pneumonia (n = 240), bronchopleural fistula (n = 29), others (atelectasis, pleural effusion, etc.) (n = 453). Patients could have more than one complication

^d^Excludes 50 patients with unknown information

### Characteristics of patients with CPA and details of treatment

The detailed clinical characteristics of 93 patients who developed CPA are summarized in Additional file 1: Table S1. In most cases, the *Aspergillus* species was confirmed by a serologic test. Chronic cavitary pulmonary aspergillosis was the most common type (74.2%), followed by subacute invasive aspergillosis (20.4%). Of all 93 patients, 22 (23.7%) did not receive antifungal drugs, and of 71 patients who received antifungal drugs, only 45 patients were able to complete the antifungal treatment for a median of 11.1 (IQR, 6.0–16.7) months.

### Effect of CPA on mortality

Patients were followed up for a median of 5.01 (IQR, 3.41–6.70) years to assess survival, and the 5-year survival rate was 76.8% (Fig. 2B). The OS of patients who developed CPA was 90.3%, 76.3%, 62.8%, and 48.4% at 1, 3, 5, and 10 years, respectively. In a crude model, development of CPA showed an unfavorable effect on OS with a hazard ratio (HR) of 1.69 (95% confidence interval [CI], 1.23–2.32, *p* = 0.001) (Table 2). However, the effect of CPA on mortality was reversed in models 1 to 3 through additional adjustment of several variables. Finally, in model 3, the adjusted HR of CPA on mortality was 0.73 (95% CI 0.53–1.03, *p* = 0.060).

**Table 2:** Association between overall survival and development of CPA (n = 6777)

Models	Overall survival for CPA (+) (vs. CPA (–))		
	HR	95% CI	P
Crude	1.69	1.23–2.32	0.001
Model 1	1.10	0.80–1.51	0.565
Model 2	0.92	0.66–1.25	0.560
Model 3	0.73	0.53–1.03	0.060

Model 1 was adjusted for clinical baseline characteristics (age, sex, body mass index, smoking status, and comorbidities: history of pulmonary tuberculosis, chronic obstructive pulmonary disease/asthma, interstitial lung disease, diabetes mellitus, and previous history of malignancy). Model 1 was analyzed by excluding 2 patients with missing data for smoking status

Model 2 was adjusted for variables with lung cancer-related factors (tumor histology, types of surgical resection, postoperative pulmonary complications within 30 days, and neoadjuvant and adjuvant treatment: none, chemotherapy only, radiotherapy only, and chemotherapy and radiotherapy both). Model 2 was analyzed by excluding 50 patients with missing data for adjuvant treatment

Model 3 was adjusted for all the preceding variables. Model 3 was analyzed by excluding 52 patients with missing data for smoking status or adjuvant treatment

*CPA* Chronic pulmonary aspergillosis, *HR* Hazard ratio, *CI* Confidence interval

### Effect of CPA on lung function decline

**Fig. 3:**
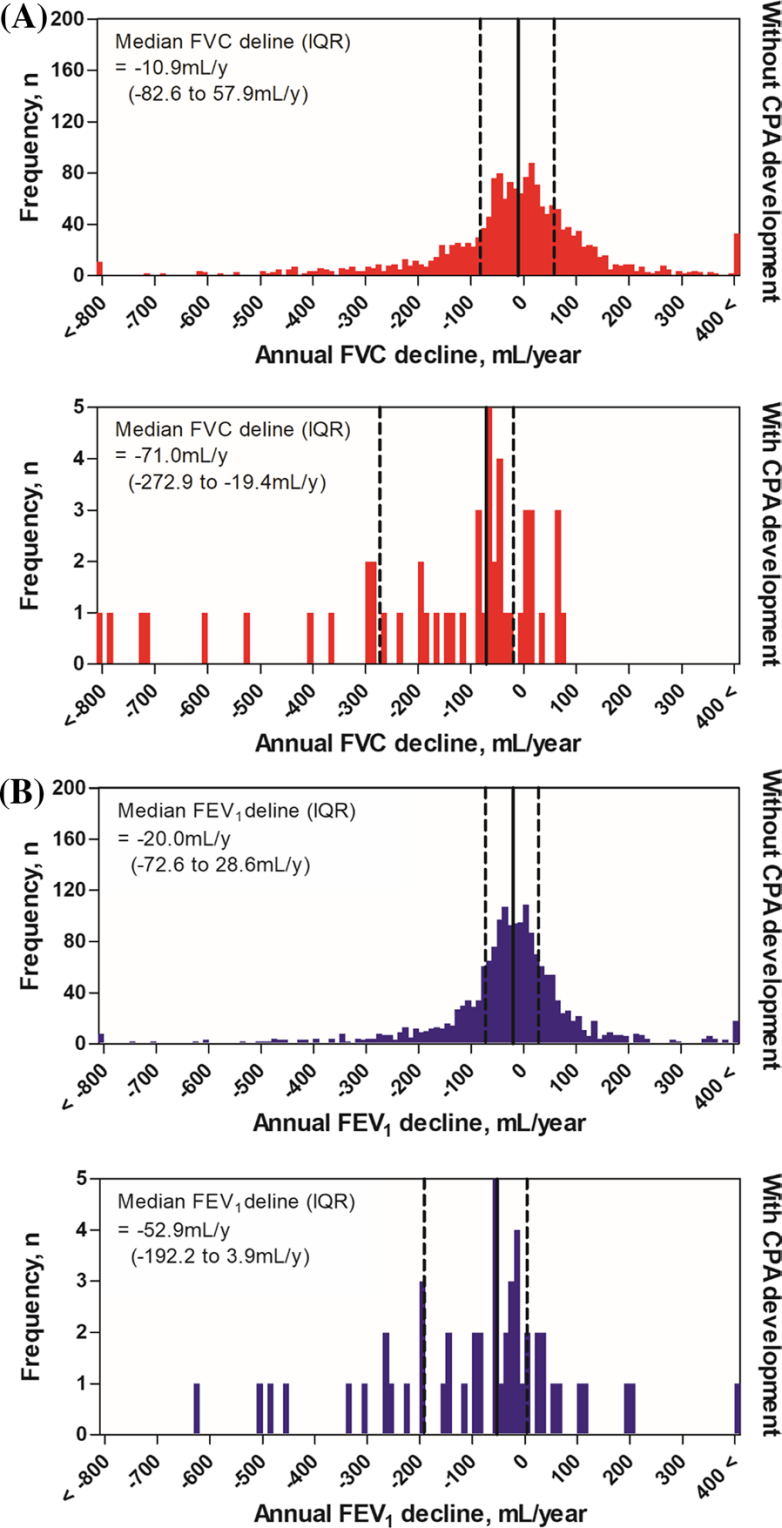
Lung function decline according to the development of CPA. Solid lines show median values, and dotted lines show interquartile ranges. **A** Histograms of annual FVC decline (mL/year) and **B** Histograms of annual FEV_1_ decline (mL/year) with or without CPA development. *CPA* Chronic pulmonary aspergillosis, *FVC* Forced vital capacity, *FEV*_1_ Forced expiratory volume in 1 s

A total of 1842 patients, 50 with CPA and 1792 without CPA, had PFT results that satisfied the criteria of this study (Table 3). At baseline, FEV_1_ [L] was not significantly different between patients with CPA and those without (1.96 vs. 2.09 L, *p* = 0.116); however, FVC [L] was lower in patients with CPA than in those without (2.70 vs. 2.97 L, *p* = 0.003). After a median of 3.7 (IQR, 1.8–4.4) years, both FVC (2.35 vs. 3.04 L, *p* < 0.001) and FEV_1_ (1.76 vs. 1.99 L, *p* = 0.001) were lower in patients with CPA than in those without. In addition, the annual declines in FVC (− 71.0 vs. − 10.9 mL/year, *p* < 0.001) (Fig. 3 A) and FEV_1_ (− 52.9 vs. − 20.0 mL/year, *p* = 0.010) (Fig. 3B) were also greater in patients with CPA than in those without. Also, the proportions of patients with rapid progression of FVC and FEV_1_ decline were higher in patients with CPA than in those without (FVC, 72.0% vs. 39.0%, *p* < 0.001; FEV_1_, 54.0% vs. 39.0%, *p* = 0.032).

**Table 3:** Comparison of lung function according to CPA development (n = 1842)

Variables	Total (n = 1842)	CPA (–) (n = 1792)	CPA (+) (n = 50)	*P*
Interval between baseline and last spirometry procedure, years	3.7 (1.8–4.4)	3.7 (1.8–4.4)	2.7 (1.5–4.7)	0.678
Baseline spirometry after surgery^a^				
FVC, L	2.96 (2.45–3.57)	2.97 (2.46–3.59)	2.70 (2.17–3.11)	0.003
FVC, % predicted	81 (68–91)	81 (69–91)	65 (54–76)	< 0.001
FEV_1_, L	2.09 (1.73–2.51)	2.09 (1.73–2.51)	1.96 (1.56–2.31)	0.116
FEV_1_, % predicted	77 (64–88)	77 (65–88)	64 (57–73)	< 0.001
FEV_1_/FVC	73 (66–79)	73 (65–88)	79 (70–84)	< 0.001
Baseline spirometry pattern				< 0.001
Normal	777 (42.2)	770 (43.0)	7 (14.0)	
Obstructive	520 (28.2)	508 (28.3)	12 (24.0)	
Restrictive	545 (29.6)	514 (28.7)	31 (62.0)	
Last spirometry results				
FVC, L	3.01 (2.39–3.38)	3.04 (2.41–3.39)	2.35 (1.76–2.67)	< 0.001
FVC, % predicted	80 (67–92)	81 (67–93)	58 (42–69)	< 0.001
FEV_1_, L	1.99 (1.62–2.44)	1.99 (1.63–2.45)	1.76 (1.43–2.10)	0.001
FEV_1_, % predicted	76 (63–89)	77 (63–89)	60 (45–70)	< 0.001
FEV_1_/FVC	71 (64–78)	71 (64–78)	77 (69–86)	< 0.001
Last spirometry pattern				< 0.001
Normal	744 (40.4)	739 (41.2)	5 (10.0)	
Obstructive	571 (31.0)	559 (31.2)	12 (24.0)	
Restrictive	527 (28.6)	494 (27.6)	33 (66.0)	
Changes in lung function				
FVC decline, mL/year	-13.2 (-87.1 to 56.4)	-10.9 (-82.6 to 57.9)	-71.0 (-272.9 to -19.4)	< 0.001
FVC decline ≤ -40 mL/year	734 (39.8)	698 (39.0)	36 (72.0)	< 0.001
FEV_1_ decline, mL/year	-20.4 (-73.3 to 28.5)	-20.0 (-72.6 to 28.6)	-52.9 (-192.2 to 3.9)	0.010
FEV_1_ decline ≤ -40 mL/year	725 (39.4)	698 (39.0)	27 (54.0)	0.032
Use of bronchodilator during the follow-up period	426 (23.1)	397 (22.2)	29 (58.0)	< 0.001

Data are presented as n (%) or the median (interquartile range)

*CPA* Chronic pulmonary aspergillosis, *FVC* Forced vital capacity, *FEV*_1_ Forced expiratory volume in one second

^a^Baseline spirometry was analyzed using the test results at least 3 months after surgery in consideration of sufficient recovery from postoperative pulmonary function deterioration

From baseline spirometry in patients with CPA, the proportion of patients with a restrictive pattern was higher than that of those without (62.0% vs. 28.7%, *p* < 0.001). In patients with CPA, 3 of 7 (42.9%) with a normal pattern and 4 of 12 (33.3%) with an obstructive pattern at baseline changed to a restrictive pattern at the last spirometry (Additional file 1: Fig. S1). On the other hand, in patients without CPA, only 97 of 770 (12.6%) patients with a normal pattern and 45 of 508 (8.9%) patients with an obstructive pattern at baseline changed to a restrictive pattern. Finally, the proportion of patients with a restrictive pattern remained higher in patients with CPA than in those without CPA (66.0% vs. 27.6%, *P* < 0.001).

The results of univariate and multiple linear regression analyses of lung function decline are summarized in Additional file 1: Table S2 and Table 4. The crude model showed that development of CPA affected the decline in FVC (β coefficient, − 132.5; 95% CI − 206.0 to − 58.9; *p* < 0.001) but not that in FEV_1_ (β coefficient, − 49.1; 95% CI − 105.6 to 7.4; *p* = 0.088). Even after adjustment of numerous clinical variables throughout the 5 models, patients with CPA had a greater FVC decline (model 5; β coefficient, − 103.6; 95% CI − 179.2 to − 27.9; *p* = 0.007) than those without CPA. However, the decline in FEV_1_ (model 5; β coefficient, − 14.4; 95% CI − 72.1 to 43.4; *p* = 0.626) did not show a significant difference in the development of CPA after variables were adjusted.

**Table 4:** Association between lung function decline and development of CPA (n = 1842)

Models	FVC decline (mL/year) for CPA (+) (vs. CPA (–))			FEV_1_ decline (mL/year) for CPA (+) (vs. CPA (–))		
	β coefficient	95% CI	*p*	β coefficient	95% CI	*p*
Crude	− 132.5	− 206.0 to − 58.9	< 0.001	− 49.1	− 105.6 to 7.4	0.088
Model 1	− 143.8	− 217.1 to − 70.4	< 0.001	− 55.1	− 111.1 to 0.9	0.054
Model 2	− 97.3	− 172.8 to − 21.7	0.012	− 16.9	− 74.4 to 40.6	0.564
Model 3	− 129.8	− 204.6 to − 55.0	0.001	− 40.2	− 97.1 to 16.6	0.165
Model 4	− 93.0	− 168.9 to − 17.1	0.016	− 20.3	− 78.8 to 38.1	0.495
Model 5	− 103.6	− 179.2 to − 27.9	0.007	− 14.4	− 72.1 to 43.4	0.626

Model 1 was adjusted for baseline lung function; FVC [L] at baseline for FVC decline; FEV_1_ [L] at baseline for FEV_1_ decline

Model 2 was adjusted for selected variables with *p* value < 0.20 in univariate analysis: sex, BMI, smoking status, COPD/Asthma, interstitial lung disease, baseline FVC [L], tumor histology, surgical approach (VATS or thoracotomy), type of surgical resection, adjuvant treatment (none, chemotherapy only, radiotherapy only, chemotherapy and radiotherapy both), and bronchodilator use for FVC decline; sex, BMI, smoking status, COPD/Asthma, baseline FEV_1_ [L], tumor histology, surgical approach (VATS or thoracotomy), types of surgical resection, and adjuvant treatment (none, chemotherapy only, radiotherapy only, chemotherapy and radiotherapy both) for FEV_1_ decline

Model 3 was adjusted for variables that were generally considered to influence the decline of lung function: age, sex, BMI, smoking status, COPD/Asthma, interstitial lung disease, baseline FVC [L] or FEV_1_ [L], and bronchodilator use

Model 4 was adjusted for variables related to lung cancer treatment and variables considered to be related to the development of CPA: BMI, smoking status, interstitial lung disease, tumor histology, surgical approach (VATS or thoracotomy), types of surgical resection, postoperative pulmonary complications within 30 days, and adjuvant treatment: none, chemotherapy only, radiotherapy only, chemotherapy and radiotherapy both)

Model 5 was adjusted for all the preceding variables: age, sex, BMI, smoking status, history of pulmonary tuberculosis, COPD/Asthma, interstitial lung disease, baseline FVC [L] or FEV_1_ [L], tumor histology, surgical approach (VATS or thoracotomy), types of surgical resection, postoperative pulmonary complications, adjuvant treatment (none, chemotherapy only, radiotherapy only, chemotherapy and radiotherapy both), and bronchodilator use

There were no missing data, so no patients were excluded from the multivariate analysis of the five models

*CPA* Chronic pulmonary aspergillosis, *FVC* Forced vital capacity, *FEV*_1_, Forced expiratory volume in one second, *CI* Confidence interval, *BMI* Body mass index, *COPD* Chronic obstructive pulmonary disease, *VATS* Video-assisted thoracoscopic surgery

## Discussion

After lung cancer surgery, 93 of 6777 patients developed CPA at a median of 3 years. The OS was lower at 5 years in patients with CPA than in those without (62.8% vs. 77.0%). However, the development of CPA did not significantly affect mortality in multivariate analysis. Meanwhile, a restrictive pattern was dominant in patients with CPA, and the FVC decline was greater in patients with CPA than in those without, even after adjustment for possible confounders.

CPA is known to rarely occur in healthy lungs, but most commonly develops in a pre-existent bronchopulmonary (or less usually, pleural cavity) disorder such as sequelae of mycobacterial diseases, chronic obstructive pulmonary disease, prior pneumothorax, treated lung cancer, fibrocystic sarcoidosis, and pneumoconiosis [11]. Our cohort has structural problems of the lungs and immunosuppression problems related to chemotherapy and/or radiotherapy, so the pathophysiology for CPA development seems to be sufficient. In addition, as the treatment outcomes for lung cancer are improving, chronic infectious disease such as CPA will eventually become a bigger problem than now. However, since there is still very little data on this, we performed these analyses with our cohort and we believe this is the novelty of our study.

A few studies have investigated the mortality and risk factors of CPA. The survival rates in CPA patients in those studies varied widely, which might have resulted from different sample sizes, underlying comorbidities, and CPA subtypes [24–28]. A study with a relatively large cohort of 387 CPA patients who were referred to the National Aspergillosis Center in the UK, reported that survival rates were 86%, 62%, and 47% at 1, 5, and 10 years, respectively [25]. However, in the study, only about 20% of the patients were lung cancer survivors, and there was no specific information about lung cancer treatment. The effect of CPA on mortality in lung cancer patients, particularly those who have undergone lung cancer surgery, is rarely reported. Tamura et al. reported the incidence and prognosis of CPA in patients who underwent lobectomy for lung cancer [13]. However, the 1-year survival rate of their study (47.0%) was lower than that in our study (90.3%). The poorer outcome of their study was possibly affected by the smaller sample size (CPA patients/total study population, 17/475 patients), older age (median 68 years), less recent data (conducted between 2000 and 2009), and inclusion of only lobectomy cases in comparison to our study. In addition, their result did not describe the clinical information of the patients including CPA subtypes and pre- or postoperative treatment. Factors not presented in the study might differ from those in our study, and these differences might have affected mortality on both studies. Future studies conducted in diverse population groups on the effects of CPA in lung cancer patients who underwent surgery are needed to build more robust evidence.

In this study, multivariate analyses of the effect of CPA on OS in the crude model indicated higher mortality in patients with CPA than in those without (HR 1.69, *P* = 0.001), but when several variables were adjusted, the HRs were opposite that of the crude model (model 3; HR 0.73, *P* = 0.060). We interpret that the development of CPA might lead to these unexpected consequences for mortality due to the moderating effect between some variables. In previous studies, variables that could affect the development of CPA were lower BMI, ever smoker, underlying interstitial lung disease (ILD), and PPC [7, 13]. However, these variables can also influence the poor OS of lung cancer patients after surgery [29, 30]. Therefore, we suggest that the effect of CPA on OS decreased as these variables were adjusted because they affected both survival analysis and CPA development, that is, they were more common in patients with CPA than in those without.

In our study, the restrictive pattern was more dominant in patients with CPA at both baseline and the last PFTs compared to patients without CPA. In addition, patients with CPA had lower FVC and FEV_1_ values at both baseline and last PFTs and had significantly greater FVC decline than those without. A restrictive pattern of spirometry is associated with space-occupying lesions and ILD [31]. The patients with CPA had more frequent ILD, history of thoracotomy, and PPC than those without CPA, which were common factors in the development of CPA and the restrictive pattern [7]. Therefore, it seems natural that patients with CPA after lung cancer surgery showed restrictive patterns at both baseline and the last PFTs. CPA itself might contribute to the restrictive patterns and FVC decline as it is a slow-progressing disease that destroys the lung parenchyma.

A recent study conducted with the general population using a national dataset reported that patients with a restrictive pattern related to poor quality of life (QOL) and lower high-intensity physical activity compared with patients with normal spirometry [32]. Thus, CPA development after lung cancer surgery might be associated with impaired QOL and low physical activity due to restrictive pulmonary function. One Chinese study with a general population cohort reported that impaired pulmonary function was significantly associated with future decreased QOL [33]. In addition, Al-Shair et al. assessed lung function and patient-reported outcome (PRO) in patients with CPA and reported that fatigue, dyspnea, and poor lung function were strongly associated with impaired health status on multivariate analysis [34]. Therefore, the development of CPA after lung cancer surgery leads to worsening of lung function that can be associated with the low QOL in lung cancer patients.

This study has several limitations. First, this is a study of a single institution and there might have been a selection bias. Second, Republic of Korea is a country with an intermediate incidence of tuberculosis, which is one of the predisposing factors for CPA [35], and this might limit the generalization of our results. Third, only 30% (2026/6684) of patients without CPA and 57% (53/93) of patients with CPA had PFT clinical information. Follow-up PFTs after lung cancer surgery were not routinely performed in patients with little or no dyspnea and occasionally could not be performed in patients with a poor general condition. If the PFTs were conducted in all patients, lung function decline would have shown a more marked difference between patients with CPA and those without. Lastly, PRO data were not obtained in our study population. If there were serially measured PROs with PFT results, the effect of CPA development on the QOL should have been clearer. Despite the limitations, to the best of our knowledge, this is the first study to report the effects of CPA on lung function decline and survival outcomes in lung cancer patients who underwent lung cancer surgery. It was also noteworthy that we analyzed a large number of patients with a long-term follow-up period.

## Conclusion

CPA is a chronic pulmonary infectious disease that can occur during follow-up after lung cancer resection surgery. Although the development of CPA after lung cancer surgery did not affect the OS, it could unfavorably contribute to the decline in lung function. Since the diagnosis of CPA is not simple and can be overlooked, patients who have risk factors for CPA after surgery should be monitored carefully for the development of CPA and managed by expert pulmonologists.

## Supplementary Information

**Additional file 1.** Online Supplement.

## Data Availability

The data of this study are available from the corresponding author upon reasonable request.

## Abbreviations

BMI: Body mass index
CI: Confidence interval
COPD: Chronic obstructive pulmonary disease
CPA: Chronic pulmonary aspergillosis
FEV_1_: Forced expiratory volume in 1 s
FVC: Forced vital capacity
HR: Hazard ratio
ILD: Interstitial lung disease
IQR: Interquartile range
NSCLC: Non-small cell lung cancer
OS: Overall survival
PFT: Pulmonary function test
PPC: Postoperative pulmonary complications
PRO: Patient-reported outcome
QOL: Quality of life
