# Supplementary material for: Mortality and lung function decline in patients who develop chronic pulmonary aspergillosis after lung cancer surgery
Source: BMC Pulm Med. 2022 Nov 22;22:436. doi: 10.1186/s12890-022-02253-y (PMC9682797; doi:10.1186/s12890-022-02253-y)
Supplement: Supplementary file 1 — Additional file 1. Online Supplement. [file 12890_2022_2253_MOESM1_ESM.docx]

**Online Supplement**

**Mortality and lung function decline in patients who develop chronic pulmonary aspergillosis after lung cancer surgery**

Bo-Guen Kim^1*^, Yong Soo Choi^2*^, Sun Hye Shin^1^, Kyungjong Lee^1^, Sang-Won Um^1^, Hojoong Kim ^1^, Yeong Jeong Jeon^2^, Junghee Lee^2^, Jong Ho Cho^2^, Hong Kwan Kim^2^, Jhingook Kim^2^, Young Mog Shim^2^, Byeong-Ho Jeong^1^

^1^Division of Pulmonary and Critical Care Medicine, Department of Medicine, Samsung Medical Center, Sungkyunkwan University School of Medicine, Seoul, Republic of Korea

^2^Department of Thoracic and Cardiovascular Surgery, Samsung Medical Center, Sungkyunkwan University School of Medicine, Seoul, Republic of Korea.

^*^Kim B-G and Choi YS contributed equally to this work.

**Correspondence to**: Byeong-Ho Jeong, M.D., PhD

Division of Pulmonary and Critical Care Medicine, Department of Medicine, Samsung Medical Center, Sungkyunkwan University School of Medicine, Irwon-ro 81, Gangnam-gu, Seoul 06351, Republic of Korea.

Tel: (+82) 02-3410-3429; Fax: (+82) 02-3410-3849; E-mail: myacousticlung@gmail.com

**Running head:** Clinical course of developed CPA after lung cancer surgery

**(A)**


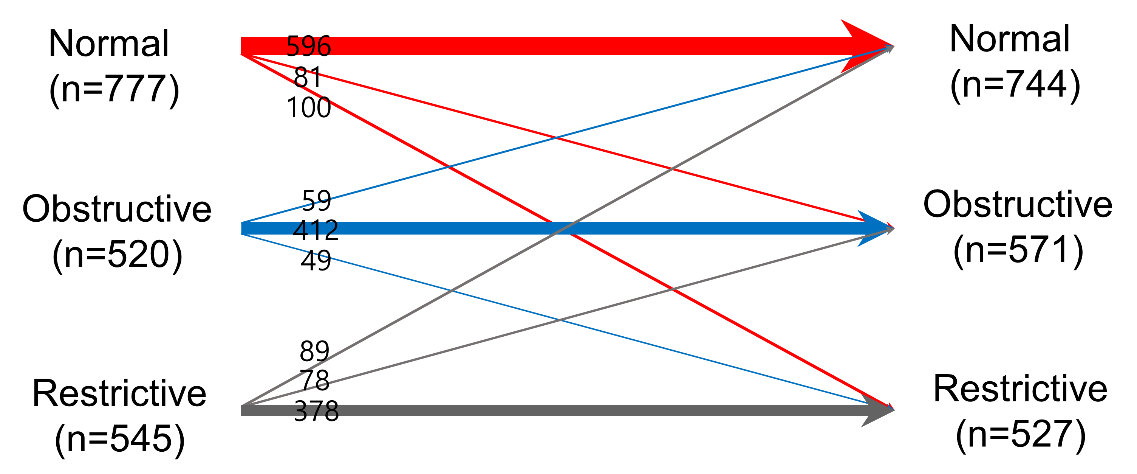


**(B)**

**
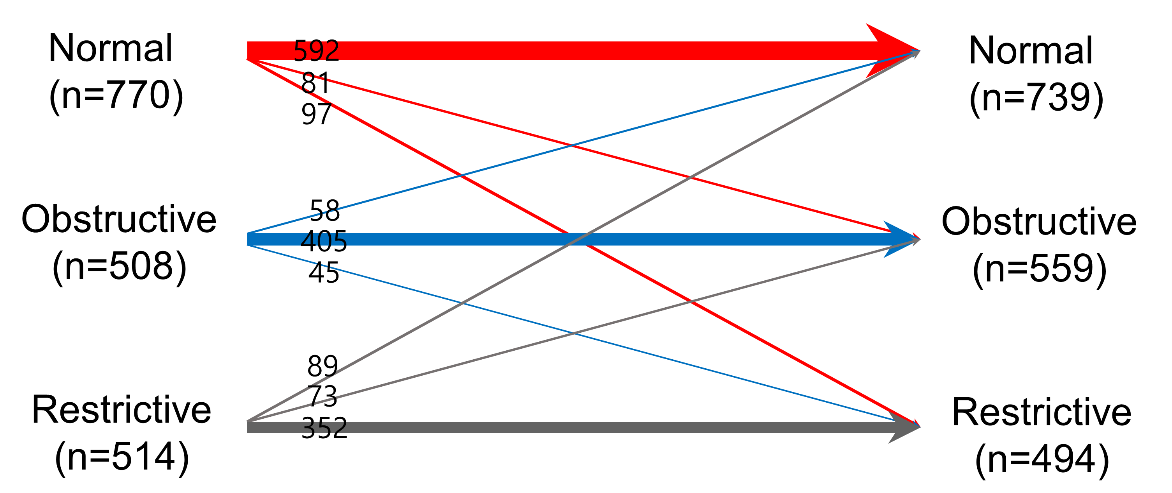
**

**(C)**

**
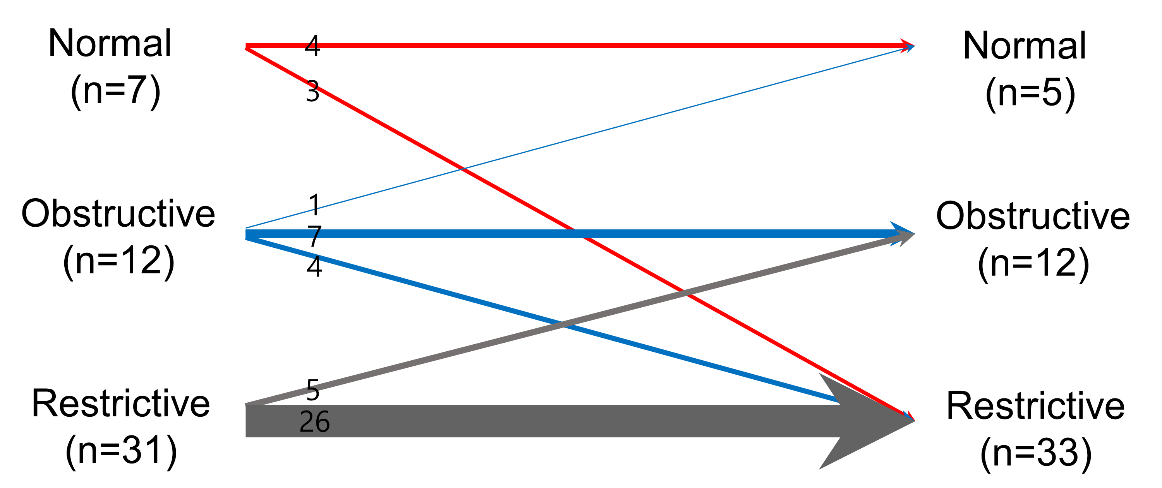
**

**Supplementary Figure 1.** Changes of baseline and last spirometry patterns according to the development of CPA. (A) Total patients. (B) Patients without CPA. (C) Patients with CPA.

An obstructive pattern is indicated by FEV_1_/FVC < 70% and FEV_1_ (% predicted) < 80%; a restrictive pattern is indicated by FEV_1_/FVC ≥ 70% and FVC (% predicted) < 80%; and a normal pattern is indicated by FEV_1_/FVC ≥ 70% and FVC (% predicted) ≥ 80% or FEV_1_/FVC < 70% and FEV_1_ (%predicted) ≥ 80%.

**Supplementary Table 1.** Detailed information of CPA after lung resection (n = 93)

| Variables | n = 93 |
| --- | --- |
| Confirmation method of *Aspergillus* spp*.* |  |
| Serology (+) for *Aspergillus* IgG | 91 (97.8) |
| Culture (+) from biopsy specimen | 2 (2.2) |
| Type of CPA ^a^ |  |
| Aspergilloma | 4 (4.3) |
| CCPA | 69 (74.2) |
| CFPA | 1 (1.1) |
| SAIA | 19 (20.4) |
| CPA involvement side |  |
| Ipsilateral side of the resected lung | 79 (84.9) |
| Contralateral side of the resected lung ^b,c^ | 9 (9.7) |
| Bilateral ^b^ | 5 (5.4) |
| Duration of antifungal treatment ^d^ | 6.0 (2.1–12.1) |
| None ^e^ | 22 (23.7) |
| Cessation of treatment ^f^ | 26 (28.0) |
| Treatment duration, months | 1.3 (0.4–2.4) |
| Treatment completed | 45 (48.4) |
| Treatment duration, months | 11.1 (6.0–16.7) |
| Antifungal drugs used (n = 71) |  |
| Itraconazole | 52 (73.2) |
| Voriconazole | 13 (18.3) |
| Itraconazole → voriconazole ^g^ | 6 (8.5) |

Data are presented as n (%) or the median (interquartile range).

^a^ Aspergilloma is defined as single pulmonary cavity containing a fungal ball with serological or microbiological evidence implicating *Aspergillus* spp. in a non-immunocompromised patient with minor or no symptoms and no radiological progression over at least 3 months of observation. CCPA is defined as one or more pulmonary cavities possibly containing one or more aspergillomas or irregular intraluminal material, with serological or microbiological evidence implicating *Aspergillus* spp. with significant pulmonary and/or systemic symptoms and overt radiological progression over at least 3 months of observation. CFPA is defined as severe fibrotic destruction of at least two lobes of lung complicating CCPA. SAIA is defined as invasive aspergillosis indicating a positive *Aspergillus* galactomannan antigen in blood, but in mildly immunocompromised patients, occurring over 1–3 months.

^b^ These 14 patients had the following structural problems in the contralateral lung at the time of lung cancer surgery: Sequelae of pneumonia (n = 5), non-tuberculous mycobacterial pulmonary disease (n = 4), bronchiectasis (n = 1), chronic obstructive pulmonary disease with emphysematous lung (n = 1), history of tuberculosis (n = 1), idiopathic pulmonary fibrosis (n = 1), another cavitary lung cancer (n = 1).

^c^ One patient underwent pneumonectomy for treatment of lung cancer and CPA developed in the remaining contralateral lung.

^d^ Excluded 22 patients not receiving antifungal treatment.

^e^ Reasons for not receiving treatment include deterioration of general condition (n = 10), no symptoms and stable lesion (n = 7), concerns about drug interactions with rifampicin or rivaroxaban (n = 4), and follow-up loss (n = 1).

^f^ Reasons for short treatment duration include death during antifungal treatment (n = 12), drug side effects (n = 8), rapid improvement of lesion during treatment (n = 3), and follow-up loss (n = 3).

^g^ Due to failure of itraconazole treatment.

CPA, chronic pulmonary aspergillosis; CCPA, chronic cavitary pulmonary aspergillosis; CFPA, chronic fibrosing pulmonary aspergillosis; SAIA, subacute invasive aspergillosis.

**Supplementary Table 2.** Univariate linear regression analysis of lung function decline (n = 1842)

| Variables | FVC decline, mL/year | | | FEV_1_ decline, mL/year | | |
| --- | --- | --- | --- | --- | --- | --- |
|  | β coefficient | 95% CI | *P* | β coefficient | 95% CI | *P* |
| Age, years | -0.9 | -2.2 to 0.5 | 0.209 | 0.54 | -0.5 to 1.6 | 0.300 |
| Sex, male | -40.3 | -64.8 to -15.8 | 0.001 | -28.4 | -47.2 to -9.5 | 0.003 |
| BMI, kg/m^2^ | 3.5 | -0.6 to 7.6 | 0.096 | 4.0 | 0.8 to 7.1 | 0.013 |
| Smoking status, yes | -32.3 | -56.5 to -8.2 | 0.009 | -20.3 | -38.8 to -1.8 | 0.032 |
| COPD/Asthma | -47.7 | -74.2 to -21.1 | <0.001 | -15.3 | -35.7 to 5.1 | 0.141 |
| History of pulmonary tuberculosis | -8.3 | -44.2 to 27.6 | 0.651 | 15.1 | -12.4 to 42.5 | 0.283 |
| Interstitial lung disease | -125.3 | -228.8 to -21.8 | 0.018 | -47.0 | -126.4 to 32.4 | 0.246 |
| Baseline spirometry |  |  |  |  |  |  |
| FVC, L | -29.8 | -44.4 to -15.2 | <0.001 | -20.9 | -32.1 to -9.7 | <0.001 |
| FEV_1_, L | -30.4 | -50.1 to -10.7 | 0.003 | -45.8 | -60.8 to -30.8 | <0.001 |
| Tumor histology |  |  |  |  |  |  |
| Adenocarcinoma | Reference |  |  | Reference |  |  |
| Squamous cell carcinoma | -42.4 | -70.6 to -14.3 | 0.003 | -24.5 | -46.2 to -2.9 | 0.026 |
| Others ^a^ | -70.7 | -132.4 to -9.0 | 0.025 | -34.6 | -82.0 to 12.7 | 0.151 |
| Surgical approach, thoracotomy | -39.9 | -65.4 to -14.4 | 0.002 | -25.4 | -45.0 to -5.8 | 0.011 |
| Types of surgical resection |  |  |  |  |  |  |
| Sublobar resection | Reference |  |  | Reference |  |  |
| Lobectomy | -12.1 | -42.8 to 18.6 | 0.438 | -3.2 | -26.7 to 20.3 | 0.788 |
| Bilobectomy | -50.7 | -110.6 to 9.2 | 0.097 | -34.6 | -80.5 to 11.3 | 0.140 |
| Pneumonectomy | -5.9 | -82.2 to 70.5 | 0.880 | -23.8 | -82.3 to 34.7 | 0.426 |
| Postoperative pulmonary complications within 30 days, yes | -13.1 | -45.4 to 19.2 | 0.426 | 0.8 | -23.9 to 25.6 | 0.946 |
| Adjuvant treatment |  |  |  |  |  |  |
| No | Reference |  |  | Reference |  |  |
| Chemotherapy only | -23.8 | -56.1 to 8.5 | 0.149 | -20.4 | -45.2 to 4.40 | 0.107 |
| Radiotherapy only | -113.1 | -178.2 to -48.0 | 0.001 | -79.8 | -129.7 to -29.8 | 0.002 |
| Chemotherapy and radiotherapy both | -29.6 | -86.0 to 26.8 | 0.303 | -24.0 | -67.2 to 19.3 | 0.277 |
| Bronchodilator use | -41.6 | -69.9 to -13.2 | 0.004 | -8.1 | -29.9 to 13.7 | 0.468 |

^a^ Includes large cell neuroendocrine carcinoma, adenosquamous carcinoma, pleomorphic carcinoma, adenoid cystic carcinoma, mucoepidermoid carcinoma, epithelial myoepithelial carcinoma, and carcinoid tumor.

There were no missing data.

FVC, forced vital capacity; FEV_1_, forced expiratory volume in one second; CI, confidence interval; BMI, body mass index; COPD, chronic obstructive pulmonary disease.
